# Modeling the effect of emergency response on domino effects in the coal gasification process by fuzzy hierarchical analysis and Bayesian network

**DOI:** 10.1371/journal.pone.0279346

**Published:** 2023-02-02

**Authors:** Liping Guo, Zhirong Wang, Dan Zhao, Kun Zhao, Pinkun Guo

**Affiliations:** 1 College of Safety Science and Engineering, Nanjing Tech University, Nanjing, China; 2 College of Petrochemical Technology, Lanzhou University of Technology, Lanzhou, China; University Campus Bio-Medico of Rome, ITALY

## Abstract

Emergency response has an important impact on the mitigation of the domino effects. However, consequence analysis of the domino effect often ignores emergency response due to its complexity and uncertainty. In this paper, Fuzzy Analytic Hierarchy Process (FAHP) is adopted to evaluate the reliability of emergency response process. On this basis, using Bayesian Network (BN) model, the domino effect under the influence of emergency response is modeled. Based on the total duration of the above-mentioned emergency response process to determine the consequences of domino effect under the action of safety barriers. The application of the approach has been demonstrated by an illustrative case study. The results show that the probability of domino effect is reduced by an order of magnitude when active barriers, passive barriers and emergency response are considered together. This work can provide relevant basis for formulating safety protection measures in chemical process industry.

## 1. Introduction

Coal gasification process is one of the most typical processes in coal chemical industry, which contains a variety of inflammable, explosive, toxic and dangerous chemicals, and is prone to accidents with serious consequences, causing great risks to people, property and the environment. Such accidents can then cause damage to the surrounding facilities, thus causing a domino effect. The protective barriers are installed at coal gasification facilities to reduce the possibility of domino events. Actually, different types of safety barriers can effectively delay or prevent the situation from escalating [1, 2]. Therefore, some scholars have done research on the performance of safety barriers in preventing domino effects, such as quantitative evaluation of safety barrier performance to mitigate fire domino effects [3–5], and emergency response to prevent escalation of accidents [6].

An emergency is a situation that poses an immediate risk to human health, life, and property, and it needs urgent interventions to prevent its deterioration. These interventions are organized as a process that is usually described in an emergency plan, called an emergency response [7]. Effective emergency response relies on a thorough integration of emergency plans at all levels of the organization, such as through using the Incident Command Systems (ICS) [8]. Rapid emergency action and effective emergency rescue have become the key to controlling the spread of accidents and reducing the harmful consequences. Inadequate emergency response capacity or poor decision-making could lead to further expansion of the accident. For instance, explosion accident occurred in Tianjin Binhai New Area, China, on Aug 12, 2015, killed 165 people. The resources, coordination and time of these actions have a great influence on the spread of industrial fire accidents or the trigger of domino effects [9]. Agent-based modeling and simulation method is applied to a major accident, which determines the allocation of limited resources and serves as an auxiliary tool for emergency response [10]. To model these relationships, multi-types of Petri nets have been used to model and analyze the emergency response [7, 11–13]. Therefore, once a fire occurs, how to do a good job of industrial fire emergency response analysis is the key to improving the efficiency of emergency response and reducing the probability of domino effect [14–18].

Both the Fuzzy Analytic Hierarchy Process (FAHP) and the Bayesian network (BN) are regarded as effective tools to solve the uncertainty in quantitative risk analysis [19]. FAHP was applied to the safety analysis of offshore oil platforms and subways, which shows the superiority of this method in system assessment [20–22]. In recent years, BN is widely used in the representation and reasoning of uncertain knowledge in risk assessment [19, 23, 24]. In order to deal with unexpected accidents, it is very important to improve the emergency response ability of the petrochemical industry. However, in the previous evaluation of consequences of the domino effect, the influence of emergency response was not considered [7]. It is worth noting that the data about the reliability of emergency response is limited, so it is a difficult task to extract the conditional probability table (CPT) of emergency response nodes in BN. In such situation, FAHP is adopted in this paper to calculate the CPT of emergency response nodes of BN.

In this paper, using FAHP to evaluate the reliability of emergency response. Establishing a comprehensive evaluation index system and evaluation model for the reliability of emergency response, realizing the systematic and comprehensive quantitative evaluation of the reliability of emergency response, while discussing the measures and suggestions to improve its reliability. Then, a BN model of domino effect is established to estimate the probability of domino effect under the influence of emergency response. The consequences of domino effect are calculated by burned area. Finally, a case study is conducted to validate the proposed model.

## 2. Domino effects in the coal gasification process

### 2.1. Coal gasification process

**Fig 1:**
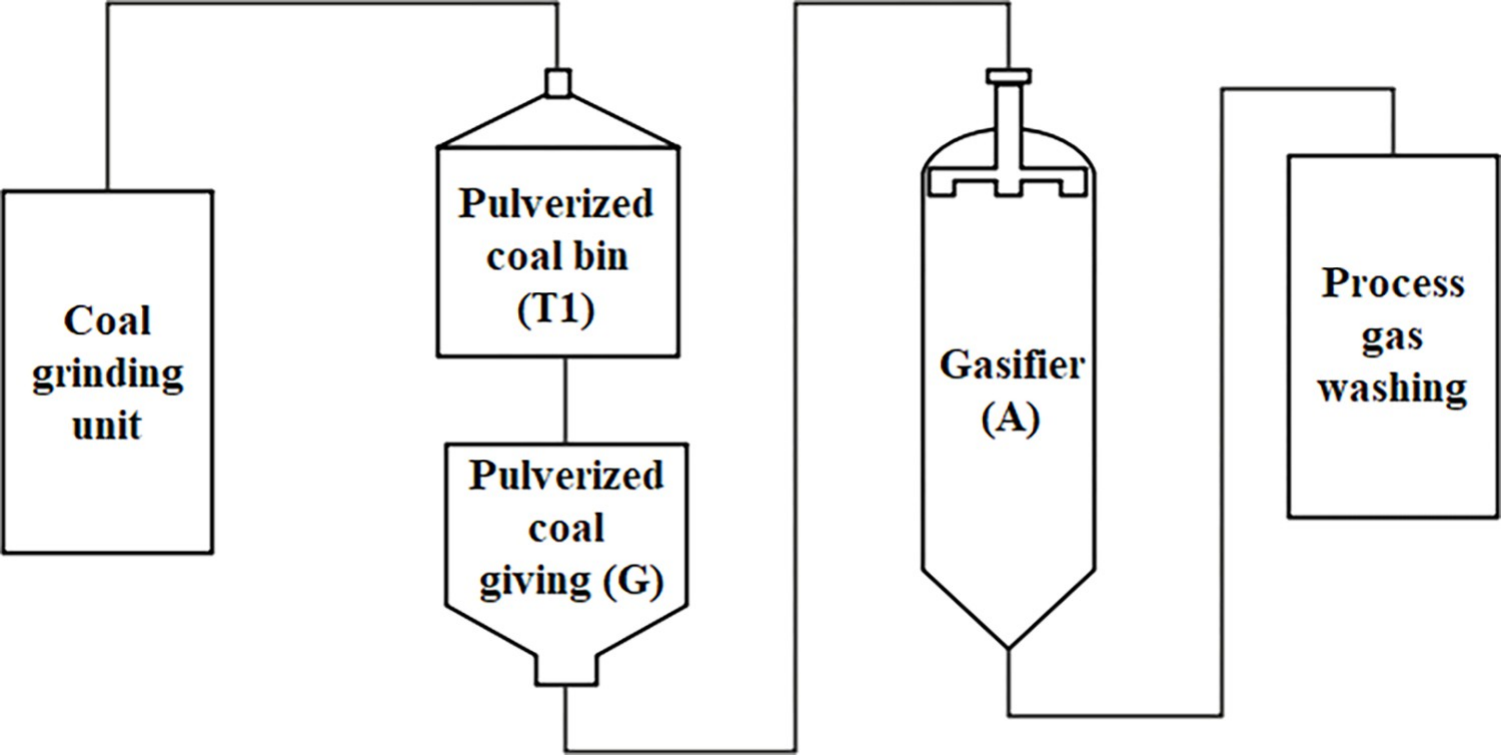
Overview of the coal gasification process.

Coal gasification process is based on coal as raw material, oxygen and water vapor as gasification agent, under the conditions of high temperature and high-pressure reaction to produce crude syngas. The crude syngas is cleaned and dusted in the washing tower. The product of coal gasification process is syngas after primary purification. The crude syngas after washing will be further synthesized. The coal gasification process flow chart is shown in Fig 1, which consists of pulverized-coal bin (T1), pulverized-coal supply tank (G), gasifier (A), preliminary purification, washing, and slag-water treatment.

### 2.2. Identification of primary accident

**Fig 2:**
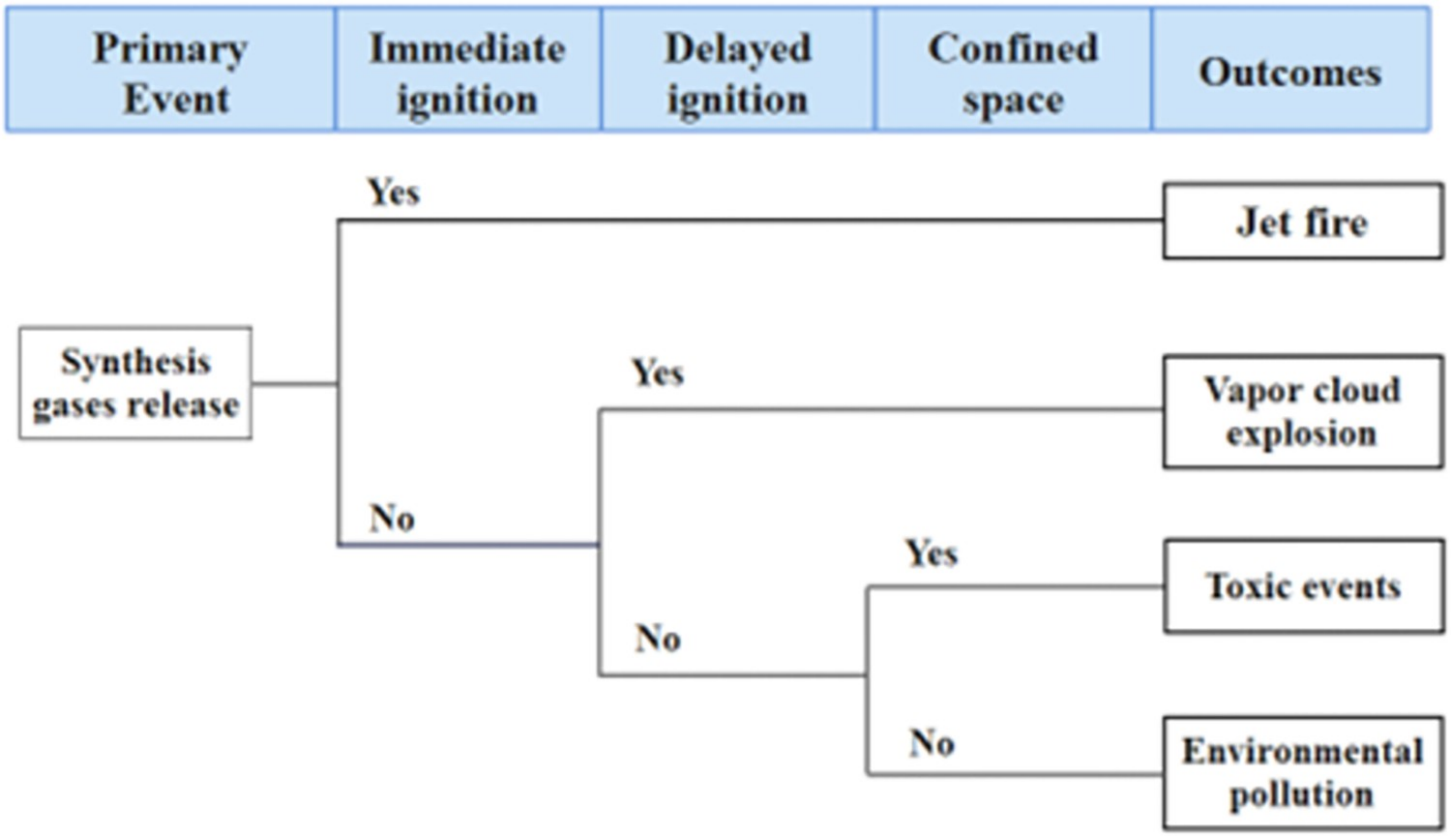
Event tree analysis of fire or explosion in the coal gasification process.

The coal gasification process has a wide variety of raw materials, intermediates and products, including various hazardous gases (CO, H_2_, CH_4_, etc.). Leakage events lead to fire or explosion events, which in turn contribute to domino effects. The Quantitative Risk Assessment Guide (Purple Book) [25] refers to material leakage inside a vessel as Loss of Containment events (LOCs). The LOCs scenarios for stationary vessels are given in Table 1. In this paper, event tree analysis (ETA) is used to identify possible primary accident, including jet fire, vapor cloud explosion, and toxic release, as shown in Fig 2.

**Table 1:** LOCs for stationary vessels.

Leakage level	Description
G.1	Instantaneous release of the complete inventory
G.2	Continuous release of the complete inventory in 10 min at a constant rate of release
G.3	Continuous release from a hole with an effective diameter of 10 mm

The release of toxic substances is always neglected since toxic substances do not cause direct damage to the target equipment. The jet fire is selected as the primary accident of synthesis gases leakage to evaluate the effect of emergency response on domino effect in the coal gasification process.

## 3. Methodology

### 3.1. Overview of AHP

AHP is a decision analysis method for evaluation, selection, ranking and prediction developed by Saaty [26]. AHP is suitable for objective systems with hierarchical interleaved evaluation indicators and for decision problems where the objective values are difficult to describe quantitatively. A four-step advance procedure is used to address the choice problem, as follows:

- Create a hierarchical model by decomposing the problem into a progressive system of choice components.
- Construct a pair-wise comparison matrix by element-based comparison.
- Check the consistency of the comparison matrix to ensure that the calculated weights are acceptable.
- Calculate the weights of choice components.

### 3.2. Fuzzy set theory

Fuzzy set theory was proposed by Zadeh [27] in 1965 for dealing with uncertainty problems. Fuzzy set is an extension of the classical representation of a set or a crisp set. Fuzzy set theory uses fuzzy numbers to represent imprecise values and adopts membership functions to describe uncertainties. In the decision-making process, fuzzy set theory is used to express the linguistic variables [28]. Linguistic variables should be converted into fuzzy numbers in order to facilitate mathematical operations. There are more types of fuzzy numbers, and the common ones are triangular fuzzy numbers, triangular fuzzy numbers, and normal-type fuzzy numbers. Among them, triangular fuzzy numbers have the characteristics of simple construction, easy operation and wide application.

In this paper, the fuzzy hierarchical analysis method is used to analyze the reliability of emergency response in coal gasification process with multi-factor. The hierarchical analysis method can quantify the decision process with less analysis, and provide a simple quantitative study of complex problems. However, in the comparison and judgment process of AHP, the analysis process is more ambiguous due to human judgment. Therefore, fuzzy theory is usually introduced to improve this process based on hierarchical analysis to form fuzzy hierarchical analysis (FAHP) [29]. FAHP has more complete, adaptable and practical results than AHP. FAHP has been widely used in risk analysis [30], risk management [31], etc.

**Fig 3:**
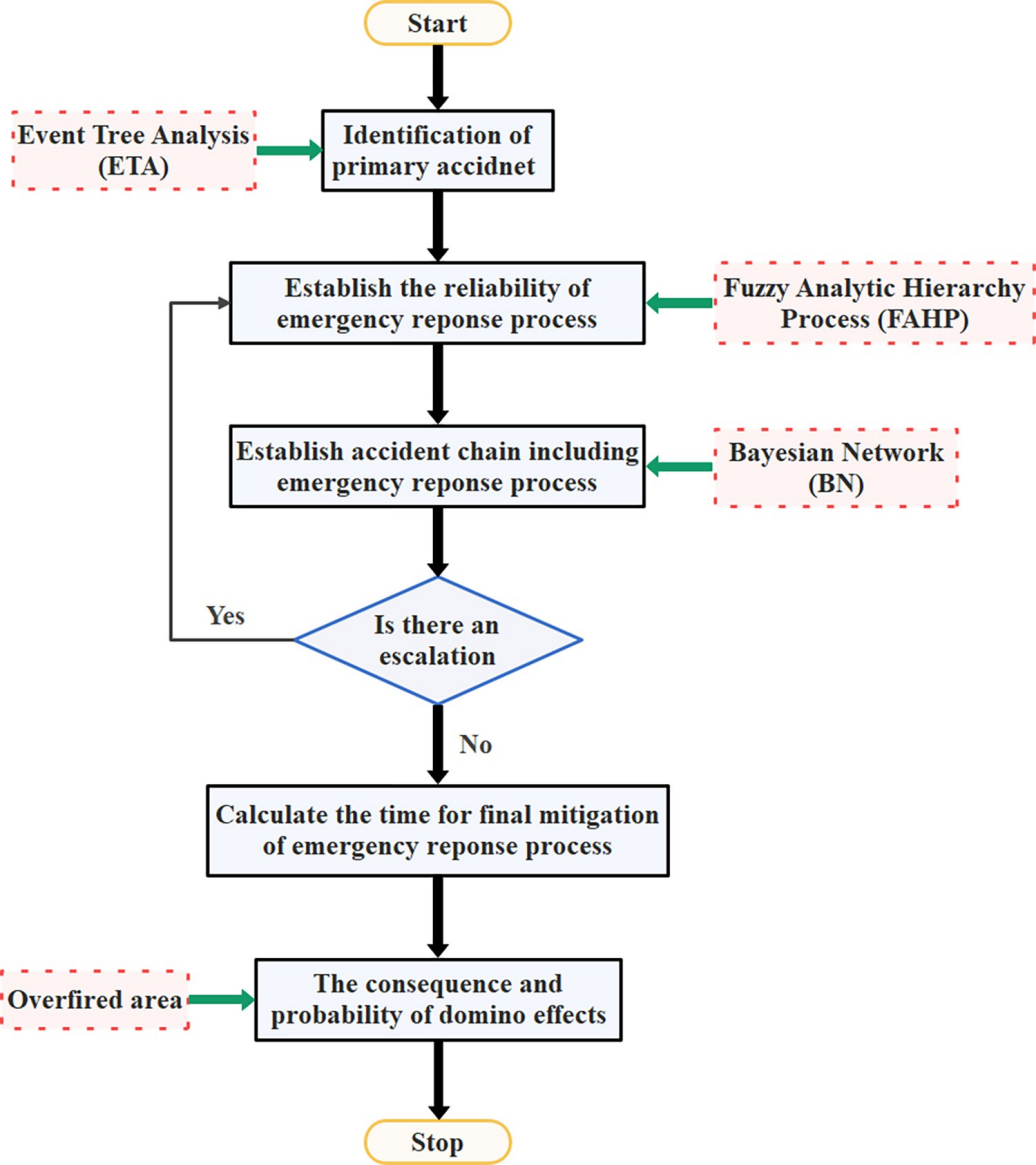
System flow diagram of the proposed method.

In this study, for the multi-factor assessment of emergency response process, the triangular degree fuzzy analysis method developed by Chang [32] is selected for research. In this model, FAHP is used to determine the reliability of different emergency response phases. Then, the domino effect chain is modeled in combination with BN. Finally, the probability and consequence (burned area) of domino accident chain under the influence of emergency response process are obtained. Fig 3 shows the flow chart of the proposed method.

## 4. Modeling reliability of emergency response

After an accident, safety protection measures have an increasing influence on the expansion of the accident, which ultimately determine the consequences of accident [33]. Through the field investigation of the coal gasification industrial park, the emergency response has the greatest influence on the mitigation of accident consequences. Therefore, this section analyzes and evaluates the reliability of emergency response.

### 4.1. Fuzzy analytic hierarchy process

#### 4.1.1. Indexes

The weighting values, which reflect the status or role of various factors in the evaluation process, directly affect the decision-making results [34]. In the AHP, developing a comprehensive, appropriate, and objective hierarchical evaluation index system is important.

**Fig 4:**
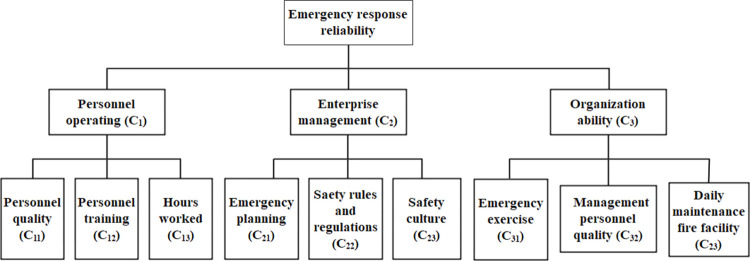
Hierarchical evaluation index system of the reliability of the emergency response. (a) Personnel operating (C_1_). In the process of emergency rescue, firefighters and safety management personnel of enterprises are the main operators, making rescue plans and launching rescues [35]. The reliability of their behaviors will affect the rescue effect to a large extent. Three sub-factors are involved in the index system, namely, personnel quality (C_11_), personnel training (C_12_), hours worked (C_13_). Personnel quality (C_11_): Numerous psychological and sociological studies have shown that the quality of personnel has a huge impact on their behavior, which can be measured by working years, education and position [36]. Personnel training (C_12_): The ability to remember and respond to events is a process that needs to be deliberately repeated. Just like the learning process, constant review deepens knowledge, ensuring that it can be applied subconsciously. However, if the interval between repeated exercises is too long, the mental attention will also form a relaxed subconscious mind, which will have a negative impact on their work [37]. Hours worked (C_13_): Please note that this factor refers to the working hours at the scene of the accident, which is different from sub-factor C_11_. Psychologists have shown that it is impossible for humans to concentrate highly for a long time, and attention will inevitably decline after a period of time [38]. Therefore, we designed the sub-factor C_13_ based on the results of this study. Emergency rescue is a very physical and mental activity, so it is influenced by hours worked. (b) Enterprise management (C_2_). After an accident occurs, the degree of control over the accident can reflect the reliability of the enterprise’s management [39]. If an enterprise has a complete safety management system, its degree of accident control will be better than other enterprises. In this factor, three sub-factors are considered, namely, emergency planning (C_21_), safety rules and regulations (C_22_), enterprise safety culture (C_23_). Emergency planning (C_21_): Emergency planning which plays the very important role in emergency response is a kind of guide document [40]. The attributes of emergency planning can be analyzed from the aspects of completeness of file system, the logicality of the content description, operability of the planning and guidance of emergency decision-making. Safety rules and regulations (C_22_): As the basic standard of an enterprise, safety rules and regulations play an important role in maintaining the normal operation of enterprises, standardizing the behavior of employees, and safeguarding their basic rights and interests. Compared with enterprises without perfect safety systems, the behavior of staff will show more appropriate handling ability and better results in the emergency rescue process. Safety culture (C_23_): Safety culture is an aspect of corporate culture, which shows tenacious vitality both in the accident prevention stage and in the post-accident response stage. In enterprises with well-rooted corporate safety culture, both the reduction of accident rate and the control of accident consequences show their advantages, so they are considered in this section. (c) Organizational capacity (C_3_). This factor refers to the comprehensive organizational evaluation of the accident enterprise and the fire department [41]. The implementation of the management system greatly affects the ability to respond to accidents, and the degree of implementation has similar effects on the emergency rescue process as the above two factors. This factor consists of three sub-factors. Emergency exercise (C_31_): Through regular actual combat simulation exercises, further improve and optimize the emergency plan, and determine the evaluation of this sub-factor according to the frequency of emergency exercises. Management personnel quality (C_32_): Please note that this factor refers to the managers who judge the accident and deploy rescue operations, which is different from the sub-factor C_11_. If the judgment is wrong, it will lead to a deviation of the command, and the emergency rescue will fail. Daily maintenance fire facility (C_33_): This sub-factor refers to the firefighting equipment inside the enterprise, such as fire alarm devices, fire hose, etc. In order to ensure the reliability of equipment, attention should be paid to its daily overhaul and maintenance work.

On the basis of combining expert opinions and literature data, this paper first establishes an evaluation index system, including three basic factors of personnel operating (C_1_), enterprise management (C_2_) and organizational capability (C_3_). Each basic factor contains a set of sub-factors. Emergency response reliability determines the severity of accident consequences, which is mainly related to the quality of the personnel involved in rescuing, the level of enterprise safety management and the ability to rescue the accident site. The sub-factors are manifestations of reliability and are influenced by personal and external factors. Therefore, there would have been no mutual influence between the sub-factors. It can be seen that both the basic factors and subfactors all meet the requirement of mutual independence. The hierarchical evaluation index system for emergency response reliability in this paper is shown in Fig 4.

### 4.1.2. Establishment of pair-wise comparison matrices

After constructing the hierarchical index system, the next step is to construct a pair of comparison matrices. The layout of these matrices makes it possible for each element in the matrix to be compared with other elements directly and individually. This comparison is done by using a numerical scale, which means that one element is more relevant or dominant than another. The matrices are constructed to compare the relative importance of each of the comparison criteria and to compare each alternative with respect to each of the comparison criteria. The fundamental scale of absolute numbers that was suggested for AHP is shown in Table 2 [26].


$$C=\left(\begin{array}{ccc} A_{11} & \cdots & A_{1n}\\ \vdots & \ddots & \vdots \\ A_{n1} & \cdots & A_{nn} \end{array}\right)$$ (1)

where *C* is the pairwise comparison matrices, *A_ij_* is a number between 1 and 9. The corresponding conversion rules are shown in Eq (2).


$$A_{ji}=\left\{ \begin{array}{c} A_{ij},A_{ij}\neq 1\\ {\left(A_{ij}\right)}^{-1},A_{ij}=1 \end{array}\right.$$ (2)

**Table 2:** Relative importance of linguistic variables to the 1–9 scale.

Linguistic variable	Intensity of importance
Equally important	1
Weak or slight	2
Moderate important	3
Moderate plus	4
Strong important	5
Strong plus	6
Very strongly important	7
Absolutely important	8
Extreme important	9

In this paper, the expert scoring method is used to determine the pairwise comparison matrices [42, 43]. In order to ensure the obtained comparison matrix is reasonable, experts from a total of three units (Research institutes, universities and companies) related to the coal gasification process are selected to express their opinions on the importance in the pairwise comparison matrix. In the design of the questionnaire, the reliability of experts’ information is firstly determined according to Table 3. The questionnaire is analyzed using the weighted results method. In this study, questionnaires are given to the above three organizations, and a total of 60 valid questionnaires are finally returned.

**Table 3:** Weight of the different expert [44].

Constitution	Classification	Score
**Professional position**	Senior academic	5
	Junior academic	4
	Engineer	3
	Assistant engineer/technician	2
	Worker	1
**Education level**	PhD	5
	Master	4
	Bachelor	3
	Higher national diploma	2
	Scholl level	1
**Experience time (year)**	≥30	5
	20–29	4
	10–19	3
	6–9	2
	≤5	1
**Age (year)**	≥50	4
	40–49	3
	30–39	2
	<30	1

The weights with the same level of importance in 60 questionnaires are added (*W_i_*). Finally, the important fuzzy number (*W_i_*) with the highest weight is selected and put into the pairwise comparison matrix. The specific calculation is shown in the following Eqs (3) and (4).


$$W_{j}=\sum_{j=1}^{4}a_{j}$$ (3)

$$W_{i}=\sum_{i=1}^{60}W_{j}$$ (4)

where *W_j_* is the weight of the expert; *a_j_* is the score of each expert according to Table 3; *W_i_* is the weight of the comparison vector.

By processing the sixty questionnaires by the above method, the comparison matrix has been finalized as shown in Tables 4–7 below. The data in Table 4 are not added due to the fact that the degree of influence of different factors is different at different stages of the emergency response process, so the comparison matrix is also different.

**Table 4:** The pair-wise comparisons of the first level factors.

	C_1_	C_2_	C_3_
C_1_	1	-	-
C_2_	-	1	-
C_3_	-	-	1

**Table 5:** Pair-wise comparisons of individual factors.

	C_11_	C_12_	C_13_
**C_11_**	1	1	1/5
**C_12_**	1	1	1/7
**C_13_**	5	7	1

**Table 6:** Pair-wise comparisons of organizational factors.

	C_21_	C_22_	C_23_
**C_21_**	1	3	1/5
**C_22_**	1/3	1	1/7
**C_23_**	5	7	1

**Table 7:** Pair-wise comparisons of group factors.

	C_31_	C_32_	C_33_
**C_31_**	1	1/5	1
**C_32_**	5	1	3
**C_33_**	1	1/3	1

### 4.1.3. Check of the consistency and the calculation of weights

The consistency of the pairwise comparison matrices can be checked by computing the consistency index (CI) and consistency ratio (CR). The CI is computed from the principal eigenvalue, *λ*_*max*_. The CR is the ratio of the CI of the pairwise comparison matrix to the average CI of a large number of reciprocal matrices of the same order whose entries are random as shown by Eq (5) [26, 45, 46].


$$CR=\frac{\left(\lambda_{\max}‐n\right)/\left(n‐1\right)}{RI}$$ (5)

where *A* is a crisp value of the fuzzy number, *a*, *b* and *c* are the triangular fuzzy numbers, respectively. *λ*_*max*_ is the largest eigenvalue, *n* is the order of the pairwise comparison matrix, and *RI* is the random consistency index chosen from Table 8.

**Table 8:** Random consistency index.

n	1	2	3	4	5	6	7	8	9
***RI***	0.00	0.00	0.58	0.90	1.12	1.24	1.32	1.41	1.45

Finally, the pairwise comparison matrix is considered consistent only if CR < 0.1. Otherwise, the parameters need to be modified until CR < 0.1. The second-order pairwise comparison matrix is consistent.

By using the eigenvector method, the weight is obtained. The values of the weight are listed in Table 9.

**Table 9:** Values of the weights, largest eigenvalue and consistency ratio.

	Crisp Weight	Largest eigenvalue	Consistency ratio
**C_11_**	0.1336	3.0126	0.0036
**C_12_**	0.1194		
**C_13_**	0.7470		
**C_21_**	0.1884	3.0649	0.0559
**C_22_**	0.0810		
**C_23_**	0.7306		
**C_31_**	0.1562	3.0291	0.0251
**C_32_**	0.6586		
**C_33_**	0.1852		

### 4.1.4. Establishment of fuzzy evaluating vector

**Fig 5:**
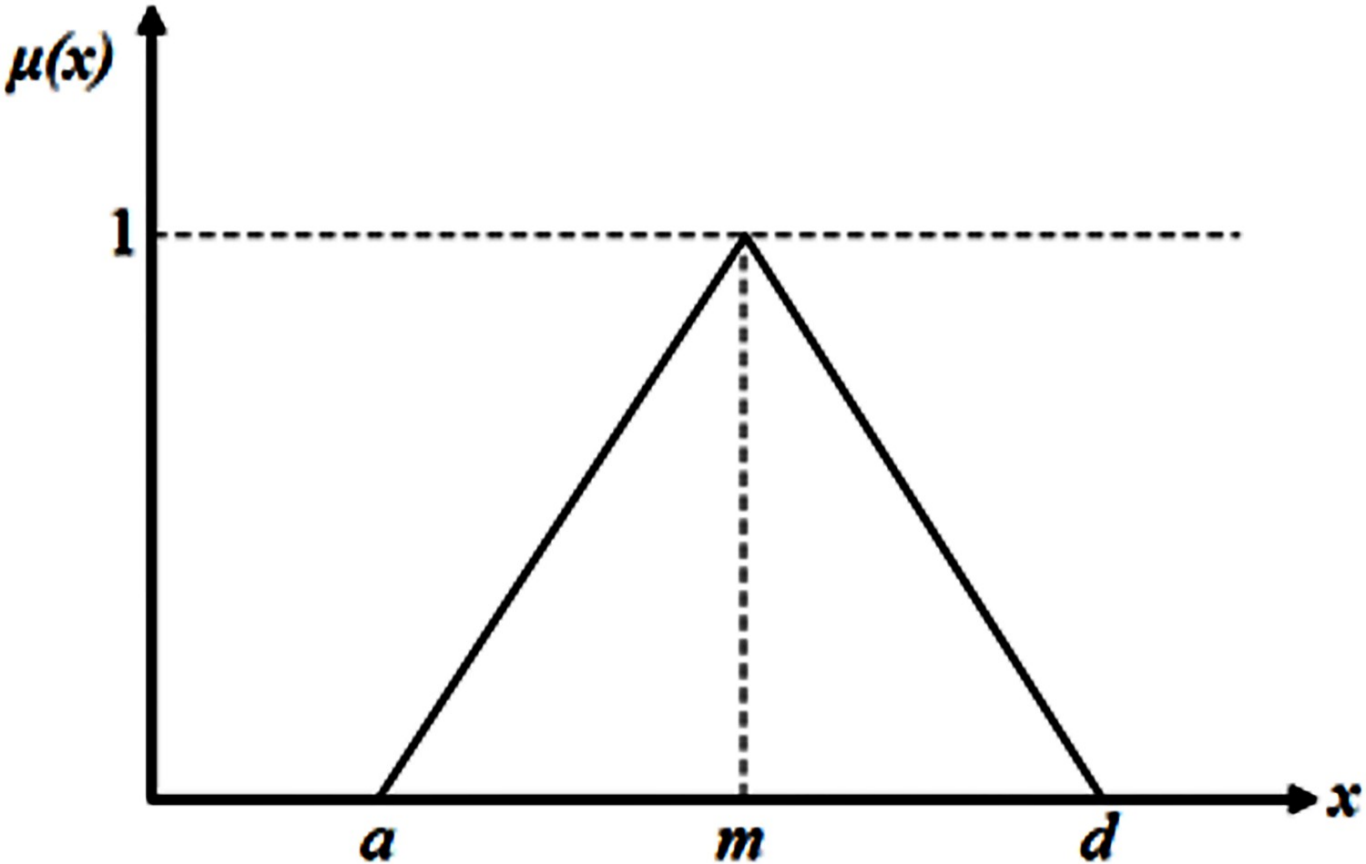
A triangular fuzzy number.

In this paper, the reliability grades for factors and sub-factors are divided into three levels: high (H), moderate (M) and low (L). In order to avoid subjective preferences and knowledge limitations of the decision group in evaluating subfactors, the reliability rating criteria are determined based on the impact of the subfactors themselves on emergency response. Before conducting the fuzzy evaluation, the numerical region of each subfactor needs to be determined. According to the actual situation of the evaluated object, the input value of each subfactor is determined, and the reliability level corresponding to its index is determined against the parameter ranges of different levels of reliability. Based on the statistical analysis of the emergency rescue process, it is determined that the interval of emergency response reliability is [10^-3^, 1] [2], and in order to perform the calculation, the corresponding linguistic variables need to be converted into fuzzy numbers (see Table 10). In this paper, the triangular extent fuzzy analysis method developed by Chang [32] is selected for research and analysis (see Fig 5).

**Table 10:** Linguistic variables that present three propagation levels to the fuzzy numbers.

Linguistic	Range	Triangular fuzzy number
**High**	10^-1^≤*P*_*extend*_<10°	(-1, -0.5, 0)
**Moderate**	10^-2^≤*P*_*extend*_<10^-1^	(-2, -1.5, -1)
**Low**	10^-3^≤*P*_*extend*_<10^-2^	(-3, -2.5, -2)

On the basis of the factor analysis (Section 4.1.1) and current research findings, the three criteria reliability grades for nine sub-factors and their associated parameter ranges are shown in Table 11 [44, 47, 48]. Comparing the collected parameter (subfactor) data with the parameter ranges for each reliability class classification (see Table 11), the fuzzy evaluation numbers of the subfactors can be obtained.

**Table 11:** Parameter ranges corresponding to the three levels of emergency response.

Sub-factors	Reliability level		
	Low (L)	Moderate (M)	High (H)
**Personnel quality C_11_**	<4	[4,6]	>6
**Personnel training C_12_**	1 time/year	[2,3] times/year	>3 times/year
**Hours worked C_13_**	>8h	[6,8]h	<6h
**Emergency planning C_21_**	No	Yes but are not comprehensive	Yes and very comprehensive
**Safety rules and regulations C_22_**	Other	Sound and less than 5 violations/month	Sound and no violations
**Enterprise safety culture C_23_**	No	Yes	Yes and hold safety activities
**Emergency exercise C_31_**	1 time/year	[2,3] times/year	>3 times/year
**Management personnel quality C_32_**	<8	[8,13]	>13
**Daily maintenance fire facility C_33_**	1 time/year	[2,4] times/year	>4 times/year

Personnel quality (C_11_) is the sum of the corresponding figures selected by the operator in Table 15 according to the actual situation. Personnel training (C_12_) is assessed using the training frequency of firefighters. Hours worked (C_13_) refers to the working hours of firefighters before the start of emergency response. Emergency planning (C_21_) is rated according to the degree of perfection of the emergency rescue plan of the enterprise by experts; Safety rules and regulations (C_22_), is based on the discipline of the fire department and the degree of perfection of the safety charter within the enterprise and the frequency of violations; Enterprise safety culture (C_23_) is determined according to whether the enterprise has its own safety culture within the enterprise and whether the activities are held. Emergency exercise (C_31_) is determined according to the frequency of holding; Management personnel quality (C_32_) is based on their actual situation by selecting the corresponding numbers in Table 12 and adding them together; the data of routine maintenance of daily maintenance fire facility (C_33_) comes from the frequency of overhauling the equipment used by firefighters and the safety guards within the enterprise.

**Table 12:** Fraction of the different operators [44].

Constitution	Classification	Score
**Education level**	PhD	5
	Master	4
	Bachelor	3
	Higher national diploma	2
	Scholl level	1
**Experience time (year)**	≥30	5
	20–29	4
	10–19	3
	6–9	2
	≤5	1
**Age (year)**	≥50	4
	40–49	3
	30–39	2
	<30	1

The input values of each second-level indicator are determined against the parameter ranges of different ranking factors according to the actual situation of the assessment object. The fuzzy evaluation vectors of subfactors are determined on the basis of their linguistic variables on the basis of Table 11 to determine the triangular fuzzy numbers, and then calculated according to Eq (6). The first-level indicators are calculated according to Eq (7) [21].


$$R_{C_{i}}=\sum_{i=1,j=1}^{i=3,j=2/3}w_{C_{\mathrm{ij}}}(⊗)f_{C_{\mathrm{ij}}}=\sum_{i=1,j=1}^{i=3,j=2/3}\left(w_{C_{\mathrm{ij}}}a_{C_{\mathrm{ij}}},w_{C_{\mathrm{ij}}}a_{m_{\mathrm{ij}}},w_{C_{\mathrm{ij}}}a_{d_{\mathrm{ij}}}\right)$$ (6)

$$R=\sum_{i=1}^{i=3}w_{C_{i}}(⊗)R_{C_{i}}=\sum_{i=1}^{i=3}\left(w_{C_{i}}a_{R_{C_{i}}},w_{C_{i}}m_{R_{C_{i}}},w_{C_{i}}d_{R_{C_{i}}}\right)$$ (7)

where *w_Cij_* is the crisp weight of the sub-factors, *w_Ci_* is the crisp weight of the factors, and *f_Cij_* is the corresponding triangular fuzzy evaluation vector.

The triangular fuzzy values need to be converted into clear values in the deblurring stage. According to Eq (8) [21], the fuzzy numbers are converted into matched clear values.


$$R=\frac{m+m}{2}+\frac{\left[(d‐m)‐(m‐a)\right]}{6}=\frac{a+4m+d}{6}$$ (8)

### 4.2. Bayesian network

Bayesian network (BN) is a probabilistic tool for uncertainty reasoning, in which nodes represent random variables, and directed arcs represent local conditional dependencies between parent nodes and child [49, 50]. And the nodes are represented by a conditional probability table (CPT). In domino effect, the initial accident is called a root node (no parents), and the final level of domino effect called a leaf node (no children). By using the chain rule and D-Separation criteria, the joint probability distribution of BN can be obtained:

$$P(U)=\prod_{i=1}^{n}P(\mathrm{Xi}|\mathrm{Pa}(\mathrm{Xi}))$$ (9)

where *Pa*(*Xi*) is the parent of *Xi*.

BNs are structured based on Bayes’ theorem, capable of updating the prior probability of some unknown variable when some evidence describing that variable exists. An important property of BN is the ability to update belief propagation, or the ability to update marginal probabilities, P(U) after observing other variables. The observed variables are called evidence. The posterior probability given can be updated when given evidence E.


$$P(U|E)=\frac{P(U,E)}{P(E)}=\frac{P(U,E)}{\sum_{U}P(U,E)}$$ (10)

In chemical process plants, the types of units are complex and dangerous. Once a domino effect occurs, its development process is affected by many factors. Based on the above analysis, using BN to evaluate the probability of domino effect under the influence of emergency response and active safety barriers can effectively solve the above uncertainty.

### 4.2.1. Multi-factor coupling evaluation of emergency response

Two coupling situations occur during the emergency response process, one of which is that two or more nodes of the same level simultaneously affect the node of the next level. For example, after the initial accident, the active barriers and on-site staff dialing the fire alarm both occurred, which has a decisive impact on the time for firefighters to arrive. The other refers to the need for firefighters to both suppress the initial accident and prevent the escalation of the accident during the emergency response process.

### 4.2.2. BN modeling of emergency response

In this research, considering the above coupling phenomena, a BN model of emergency response process is proposed. Active barriers and fire alarm are triggered simultaneously when the initial accident occurs, which is referred to as emergency response 1. In the reliability evaluation of emergency response at this time, personnel factor has a great influence on it. At least one path of the two child nodes can trigger the fire alarm.

**Fig 6:**
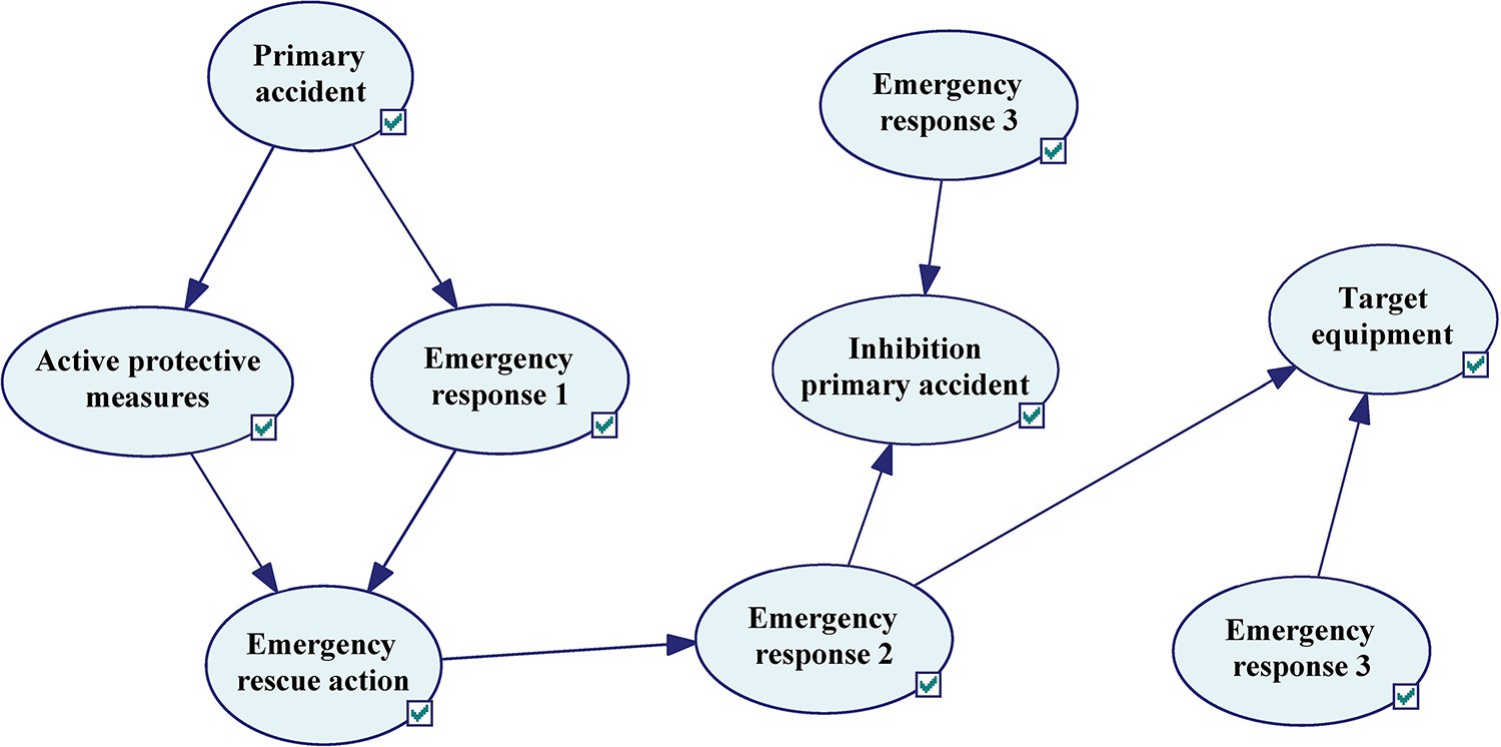
The emergency rescue process fitted by the Bayesian network diagram.

Assuming that the probability of firefighters acting immediately after receiving fire alarm is 1, the next node is the beginning of emergency rescue. After arriving at the scene, determine the target units to be suppressed and the accident to be prevented, and then completes the rescue deployment, which is called emergency response 2. At this time, the greatest impact on the reliability of the emergency response process is the completeness of the management personnel and the management system. After the work order is issued, the reliability of the emergency rescue depends more on the reliability of firefighters. Here is emergency response 3, which acts on the device being inhibited and prevented. The fitting results are shown in Fig 6.

**Fig 7:**
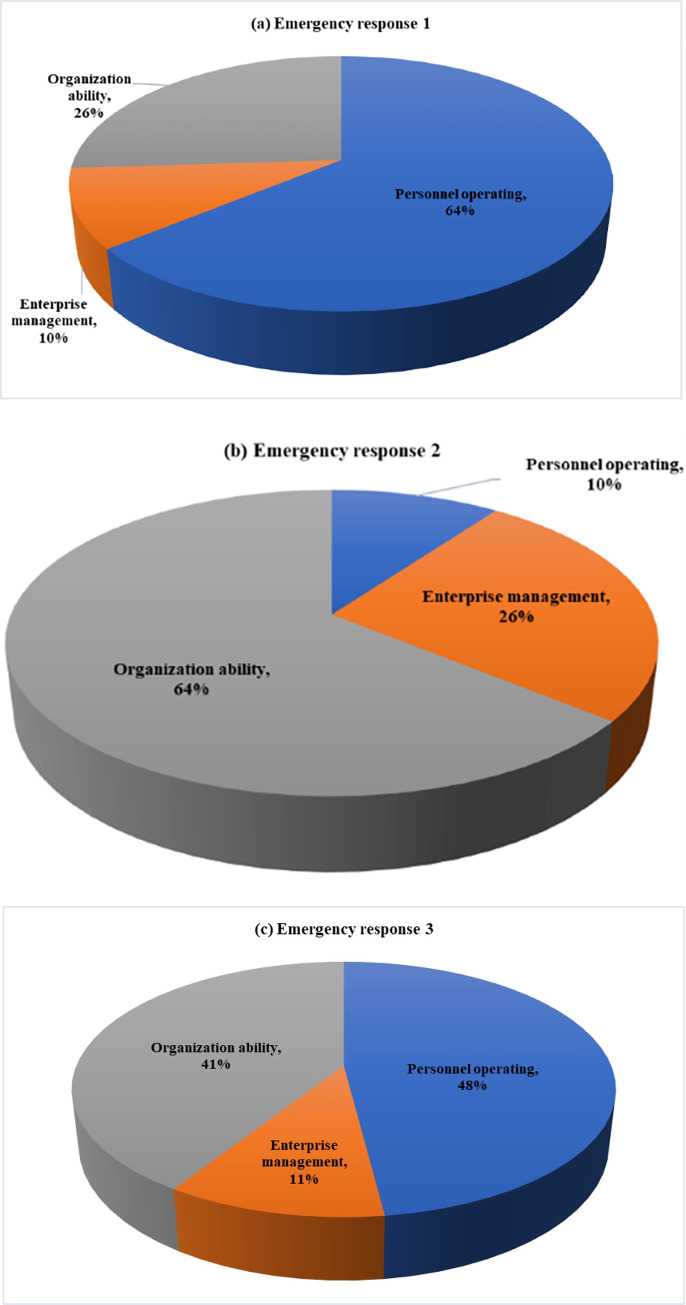
Reliability of emergency response. (a) Emergency response 1; (b) Emergency response 2; (c) Emergency response 3.

In the above process, the reliability assessment of three emergency response process appeared. Because of the different factors that had the greatest impact on them, that is, at different stages, each factor has different importance of the emergency rescue process. The comparison matrix of the analytic hierarchy process can be to solve this problem, it is enough to transform the contrast matrix of three first-level factors. Here, a comparison matrix can be established according to the relative importance of each factor at different stages of the emergency rescue process, and then the weight can be finally calculated through the consistency check (see Tables 13–18) [51–53]. The weights of first-level indicators can be seen in Fig 7 after consistency processing. For details, see 4.1.3. After obtaining its weight, the node probability is obtained by using fuzzy evaluation method, and the specific calculation method is shown in 4.1.4.

**Table 13:** Pair-wise comparisons of the first level factors in the emergency response 1.

	C_1_	C_2_	C_3_
**C_1_**	1	5	3
**C_2_**	1/5	1	1/3
**C_3_**	1/3	3	1

**Table 14:** Pair-wise comparisons of the first level factors in the emergency response 2.

	C_1_	C_2_	C_3_
**C_1_**	1	1/3	1/5
**C_2_**	3	1	1/3
**C_3_**	5	3	1

**Table 15:** Pair-wise comparisons of the first level factors in the emergency response 3.

	C_1_	C_2_	C_3_
**C_1_**	1	5	1
**C_2_**	1/5	1	1/3
**C_3_**	1	3	1

**Table 16:** Values of the consistency ratio and fuzzy weight vectors and crisp weights in emergency response 1.

	Weight	Largest eigenvalue	Consistency ratio
**C_1_**	0.6370	3.0385	0.0331
**C_2_**	0.1047		
**C_3_**	0.2583		

**Table 17:** Values of the consistency ratio and fuzzy weight vectors and crisp weights in emergency response 2.

	Weight	Largest eigenvalue	Consistency ratio
**C_1_**	0.1047	3.0385	0.0331
**C_2_**	0.2583		
**C_3_**	0.6370		

**Table 18:** Values of the consistency ratio and fuzzy weight vectors and crisp weights in emergency response 3.

	Weight	Largest eigenvalue	Consistency ratio
**C_1_**	0.4806	3.0291	0.0251
**C_2_**	0.1140		
**C_3_**	0.4054		

Among them, the probability of active barriers is calculated by combining the PFD of the early warning device and their effectiveness. More details on the application of the concepts of PFD and effectiveness (*η*) in the assessment of mitigated domino escalation are reported elsewhere [54–56]. The probability of emergency rescue action is calculated by the following Eq (11); and the probability of inhibition primary accident is calculated by the Eq (12) [56].


$$f(t_{F};\mu ,\eta )=\frac{1}{t_{F}\eta \sqrt{2\pi }}e^{-\frac{{\left[ln(t_{F})‐\mu \right]}^{2}}{2\eta^{2}}}$$ (11)

where *f*(*t_F_*; *μ*, *η*) is the probability density function of the first response time *t_F_* in city N, China; *μ* is the mean value of *ln*(*t_F_*), *μ* = 1.574; *η* is the standard deviation of *ln*(*t_F_*), *η* = 0.137.


$$f(t_{z};\mu ,\eta )=\frac{1}{t_{z}\eta \sqrt{2\pi }}e^{-\frac{{\left[ln\left(t_{z})‐\mu \right)\right]}^{2}}{2\eta^{2}}}$$ (12)

where *f*(*t_Z_*; *μ*, *η*) is the probability density function of the firefighting time *t_Z_* in city N, China; *μ* is the mean value of *ln*(*t_F_*), *μ* = 3.517; *η* is the standard deviation of *ln*(*t_F_*), *η* = 1.120.

5. Assessment of emergency response impact on consequence of domino effects

To evaluate the consequences of domino accidents, it is necessary to determine the accident chain after domino accidents are affected by the emergency rescue process, that is, determine the affected equipment. If a second-level domino accident occurs, continue the expansion to the next level until no domino accident can be caused.

### 5.1. Influencing factors

In general, the longer the accident lasts, the more hazardous material are consumed, and the more serious the accident consequences will be. From the time point of view, the inventory of hazardous material and the effectiveness of safety barriers have the greatest impact on accidents. Because of the large scale of coal gasification process and its material supply is very sufficient, this paper does not consider the impact of materials on accident consequences. On the other hand, different types of safety barriers are effective in mitigating the consequences of accidents. Three different types of barriers were identified: active barriers, passive barriers and emergency measures [3]. Therefore, this paper considers the influence of domino accidents from the above three different types of safety barriers.

### 5.2. Quantification of domino effect consequences

The consequence of domino effect is quantified by the burned areas, which is closely related to the scale of the explosion and the time of emergency response. Its size represents the severity of accident consequences, and it is positively correlated. For a fire accident, when the active measures are combined with the emergency response processes to control and prevent accidents, the action time of firefighters is determined by the actual situation of the emergency management department and enterprise. The burned area is modeled by Peng [56].


A’=1.48t+13.1 (13)

where *A*’ is the burned area under the influence of active measures and emergency response process (m^2^), *t* is the time from beginning to the end of emergency response process (min).

Besides the active measures and emergency response, passive measures also have an influence on the consequences of domino effects that cannot be ignored, which is effective from the occurrence of the accident to its own failure. In this study, the burned area is quantitatively evaluated from the reduction index and effectiveness. Eq (14) is a further optimization of Eq (13), and the final burned area can be obtained, that is, the evaluation result of the domino accident in the coal gasification unit area. The probability corresponding to the result was the data obtained in section 4.2 [57].


A=(1‐ηφ)A’ (14)

where *A* is the fire area of the domino accident under safety measures, *η* is the influence index of passive measures on the fire area, and its value is 60%, *φ* is the effectiveness of passive measures, and its value is 75%.

## 6. Case study

### 6.1. Case background

**Fig 8:**
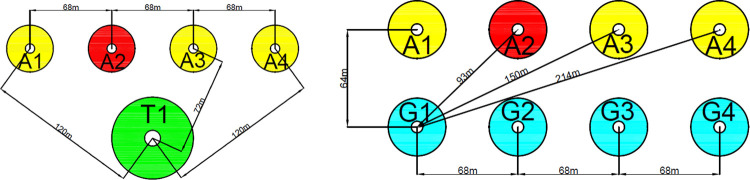
Layout of the analysis nodes in case study [58]. (a) Layout of a large silo (T1) and gasifier (A1, A2, A3, A4). (b) Layout of pulverized coal giving tanks (G1, G2, G3, G4) and gasifiers (A1, A2, A3, A4).

In this section, the proposed method is applied in the case study of Zhao [58]. The schematic of the coal gasification plant is shown in Fig 1. The characteristics of the coal gasification plant are shown in Table 19. Fig 8 shows the location relationship of the coal gasification plant.

**Table 19:** The characteristics of the device in the case study [58].

Equipment	Type	Hazardous substances	substances content (m^3^)
T_1_	Atmospheric	Coal dust	200
G_1_	Pressurized	Coal dust	120
G_2_	Pressurized	Coal dust	120
G_3_	Pressurized	Coal dust	120
G_4_	Pressurized	Coal dust	120
A_1_	Pressurized	Process gas	90
A_2_	Pressurized	Process gas	90
A_3_	Pressurized	Process gas	90
A_4_	Pressurized	Process gas	90

**Fig 9:**
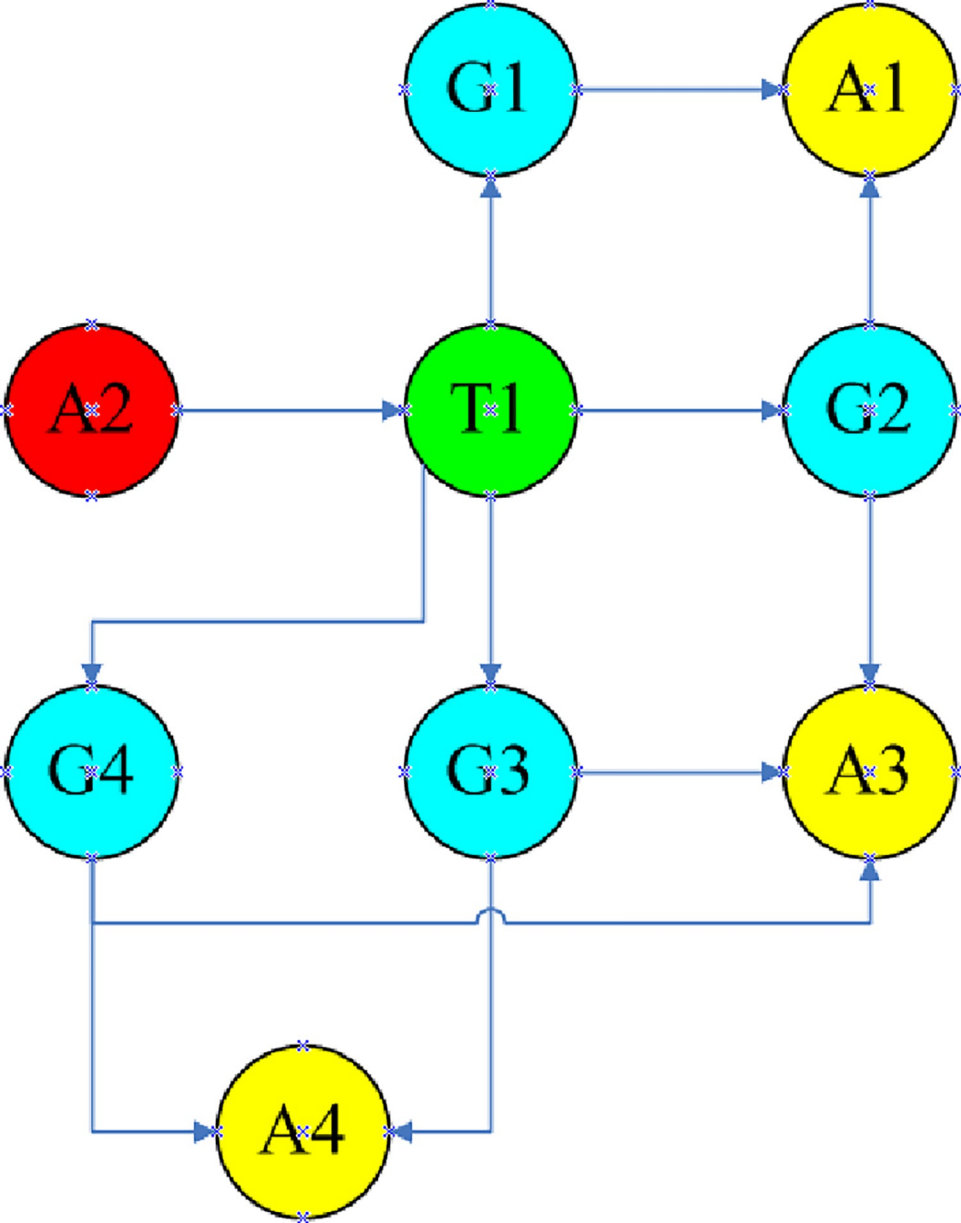
The propagation path of the case study (primary scenario in red, the second level scenario in green, the third level scenario in blue and the fourth level scenario in yellow) [58].

The thermal radiation values are compared to the threshold to determine a reasonable domino path in the case study, as shown in Fig 9. Based on this, the escalation probability of the target equipment (T1) under the influence of emergency response is updated in this case.

**Fig 10:**
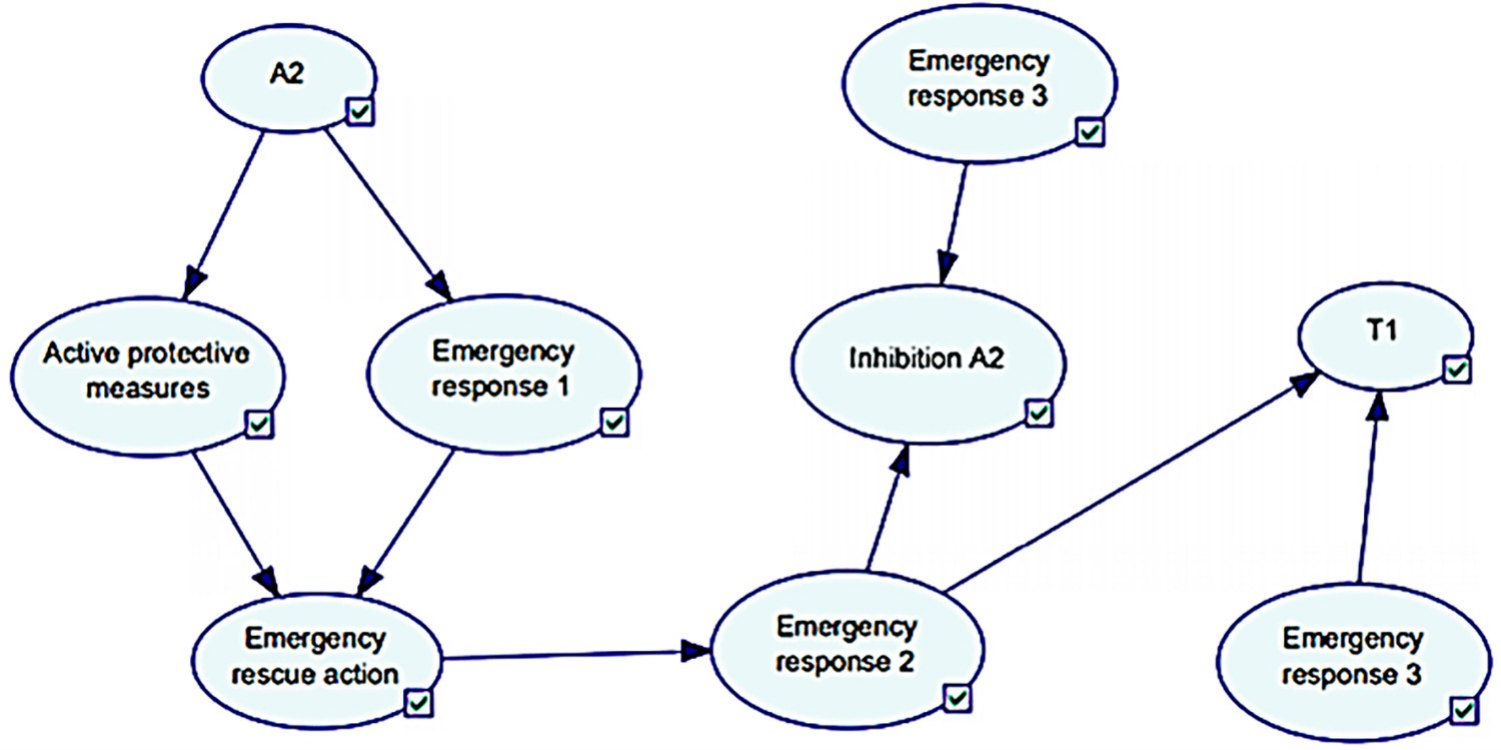
The emergency rescue process fitted by the Bayesian network diagram.

The emergency response structure of coal gasification plant consists of firefighters and safety personnel of the plant. The emergency response process has been classified into three phases based on the rescue process, namely Emergency response 1 (triggering the fire alarm), Emergency response 2 (emergency operation command) and Emergency response 3 (firefighter operation). The emergency response process fitted by the Bayesian network graph is shown in Fig 10 research and analysis.

### 6.2. Results and discussion

The reliability of three kinds of emergency rescue processes is obtained by using the above model which combines fuzzy analytic hierarchy process and fuzzy evaluation method. In this section, the reliability evaluation of the emergency rescue process is illustrated by taking the evaluation of secondary index as examples. This part of the data is determined according to the relevant situation of a coal gasification enterprise in Huainan City, Anhui Province, as detailed in Table 20.

**Table 20:** Assessment of the emergency response using FAHP.

Sub-factors	Value from case study	Reliability level	Triangular fuzzy number (Sub-factors)	Triangular fuzzy number (Factors)
**Personnel quality C_11_**	5	M	(-2, -1.5, -1)	(-1.1336, -0.6309, -0.1336)
**Personnel training C_12_**	3/year	H	(-1, -0.5, 0)	
**Hours worked C_13_**	4 hour	H	(-1, -0.5, 0)	
**Emergency planning C_21_**	Yes and very comprehensive	H	(-1, -0.5, 0)	(-1.0810, -0.5810, -0.0810)
**Safety rules and regulations C_22_**	Sound rules but violations 3–4 /month	M	(-2, -1.5, -1)	
**Enterprise safety culture C23**	Hold a safety event per half year	H	(-1, -0.5, 0)	(-1.8168, -1.3148, -0.8148)
**Emergency exercise C31**	2/year	M	(-2, -1.5, -1)	
**Management personnel quality C32**	11	M	(-2, -1.5, -1)	
**Daily maintenance fire facility C33**	6/year	H	(-1, -0.5, 0)	

According to the fuzzy evaluation method, combining the above calculation results with the weights of various factors of different emergency phases in section 4.1 the reliability of “emergency response 1”, “emergency response 2”, and “emergency response 3” can be obtained as 0.73, 0.09, and 0.13, respectively.

In this paper, only the alarm performance is considered in active measurement. A is equipped with two active measures, water deluge systems (WDS) and pressure relief valves (PRVs), which will trigger the alarm when an accident occurs. The data of its PFD and effectiveness (stable operation) are shown in Table 21 below. Then Eq (15) can be used to obtain the reliability of the active measures node is 0.999. According to the statistics and field investigation of fire alarm in China, the time of fire alarm is 5 min, and the probability calculated by Eq (11) is 0.3. Then, based on the double consideration of the total materials and fire statistics, it is determined that the time taken by firefighters from the beginning to the end to the scene is 23 min, and the probability calculated by Eq (12) is 0.22. This study provides an example of updating the CPT of emergency rescue operations in Table 22, and other CPT follows this calculation.

**Table 21:** PFD and effectiveness values of active measures [57].

Active measures	PFD	Effectiveness (դ)	
**WDS**	5.43×10^−3^	0.954	
**PRVs**	1×10^−2^	1	


$$\begin{array}{l} P_{\mathrm{AM}}=1‐[1‐\delta_{\mathrm{WDS}}(1‐{\mathrm{PFD}}_{\mathrm{WDS}})][1‐\delta_{\mathrm{PRV}}(1‐{\mathrm{PFD}}_{\mathrm{PRVs}})]\\ =1‐[1‐0.954\times (1‐5.43\times 10^{‐3})]\times [1‐1\times (1‐1\times 10^{‐2})]\\ =0.999 \end{array}$$ (15)

**Table 22:** An example of CPT of emergency rescue action.

	Emergency 1	Success		Failure	
	Active barriers	Success	Failure	Success	Failure
**Emergency rescue action**	Success	0.999	0.811	0.999	0.3
	Failure	0.001	0.189	0.001	0.7

The BN model is fitted for the first-level domino expansion process in the region of coal gasification, and the final probability of each node are calculated by the GeNie software, as shown in Table 23. It should be noted here that the accident probability of T_1_ is 2.3×10^−2^ without the emergency response process.

**Table 23:** Probability value of each node in the BN.

Nodes	Node probability	Posterior probability
**A2**	1	1
**Active barriers**	0.999	0.999
**Emergency response 1**	0.730	0.730
**Emergency rescue action**	0.300	0.996
**Emergency response 2**	0.090	0.488
**Inhibition A_2_**	0.220	0.267
**Emergency response 3**	0.130	0.130
**T_1_**	0.023 [48]	0.002

Table 23 shows that after emergency response, the expansion probability of the first-level domino accident has been reduced by one order of magnitude, to 2×10^−3^. It can be seen from the above that the firefighting operation takes 23 min, and it can be seen from the calculation of Eq (13) that the burned area of the first-level domino effect after active measures and emergency rescue is 47.14 m^2^. In the process of firefighting operation, passive measures continue to work, which can be calculated by Eq (14). After active and passive measures and emergency response, the burned area of the first-level domino effect in the coal gasification unit area is 25.93 m^2^, and the probability of occurrence is 2 ×10^−3^. Through the above calculation results, it can be concluded that the burned area caused by domino effect is reduced by 21.21 m^2^ by passive measures. The results are also shown in Table 24.

**Table 24:** Domino risk considering different types of safety barriers.

Safety barriers			Domino risk	
**Active barriers**	**Passive barriers**	**Emergency response**	**Probability**	**Consequence (m^2^)**
√	-	√	2.0×10^−3^	47
√	√	√		25.93

The results show that emergency response can effectively reduce the probability of domino effects and alleviate the consequences of domino effects. The feasibility of the model established in this paper is confirmed.

According to the results of weight calculation, in the three stages of emergency response, the most influential factors are personnel operation (0.6370), organizational capacity (0.6370) and personnel operation (0.4806). The second-level indicators that have the biggest impact on the first-level indicators are hours worked (0.7470), safety culture under enterprise management (0.7306) and management personnel quality under organizational capacity (0.6586), respectively. The results can be applied to the training and operation arrangement of rescue personnel, and targeted training can be carried out for different stages. The main measures can be summarized as follows: (1) In the stage of accident prevention, control the hours worked of safety operators, strengthen the construction of safety culture and the training of safety management personnel; (2) the rescue personnel should timely grasp the equipment that may trigger the domino effect and the danger of the affected surrounding equipment, and formulate the emergency rescue plan; (3) with regard to the layout of safety protection devices, special attention should be paid to active protection devices with alarm functions, which should be regularly inspected, maintained and replaced.

## 7. Conclusions

Based on the AHP, fuzzy evaluation method and BN, this paper establishes an evaluation model for the consequence of domino effect in coal gasification plant. In this paper, the emergency response was divided into three stages: emergency response 1 (fire alarm), emergency response 2 (emergency command) and emergency response 3 (fire-fighting operation), and the reliability were evaluated. Through field investigation, literature investigation and expert discussion, the importance of different levels of indicators in different stages of emergency response was determined, and then the reliability of emergency response was obtained. Suggestions for improving the reliability of emergency response are put forward according to the results. In the overall planning of safety protection measures, the emergency response process can be divided into three stages for consideration, and targeted improvement can be made according to the importance of each node in different stages to get twice the result with half the effort. The BN was used to simulate the emergency response process when a domino effect occurs. The results show that the probability of domino effect was one order of magnitude lower when emergency response was taken into account. Based on the above-mentioned total duration of fire-fighting operations in the emergency response process, the final burned area was determined. The analysis shows that passive fire prevention measures can greatly alleviate the consequences of domino effects.

In the current study, the influence of toxic gases on the emergency response is not considered. In fact, toxic gas will affect firefighters, which in turn affects the rescue process. Besides, in the fitting of emergency response process, it is difficult to collect early data due to the lack of domestic statistical data. Therefore, it is necessary to expand the data of personnel, management and other factors and statistics of the emergency rescue process, and establish a more accurate and complete database.

## Supporting information

S1 Table Basic information of experts. (DOCX)
