# Supplementary material for: Modeling the effect of emergency response on domino effects in the coal gasification process by fuzzy hierarchical analysis and Bayesian network
Source: PLoS One. 2023 Feb 2;18(2):e0279346. doi: 10.1371/journal.pone.0279346 (PMC10045569; doi:10.1371/journal.pone.0279346)
Supplement: S1 Table — (DOCX) [file pone.0279346.s001.docx]

**Table 1 Expert Information**

| **Expert** | **Occupational category** | **Education level** | **Experience time (year)** | **Age (year)** |
| --- | --- | --- | --- | --- |
| 1 | Senior academic | PhD | 20-29 | ≥50 |
| 2 | Senior academic | PhD | 10-19 | 40-49 |
| 3 | Senior academic | PhD | 20-29 | ≥50 |
| 4 | Senior academic | PhD | 6-9 | 30-39 |
| 5 | Senior academic | PhD | 6-9 | 30-39 |
| 6 | Senior academic | PhD | 10-19 | 30-39 |
| 7 | Senior academic | PhD | 20-29 | 40-49 |
| 8 | Senior academic | PhD | 6-9 | ＜30 |
| 9 | Senior academic | PhD | 10-19 | 30-39 |
| 10 | Senior academic | PhD | 20-29 | 40-49 |
| 11 | Junior academic | Master |  | 40-49 |
| 12 | Junior academic | Master |  | 40-49 |
| 13 | Senior academic | PhD |  | 40-49 |
| 14 | Senior academic | Master | 20-29 | ≥50 |
| 15 | Senior academic | PhD | 20-29 | ≥50 |
| 16 | Engineer | Bachelor | 10-19 | 30-39 |
| 17 | Engineer | Master | 10-19 | 30-39 |
| 18 | Senior academic | PhD | 20-29 | 40-49 |
| 19 | Assistant engineer | Bachelor | 20-29 | ≥50 |
| 20 | Engineer | Master | 20-29 | 40-49 |
| 21 | Assistant engineer | PhD | 10-19 | 30-39 |
| 22 | Senior academic | Master | 20-29 | ≥50 |
| 23 | Senior academic | Bachelor | 6-9 | ＜30 |
| 24 | Assistant engineer | Master | 10-19 | 40-49 |
| 25 | Engineer | Master | 20-29 | ≥50 |
| 26 | Engineer | Master | 10-19 | ＜30 |
| 27 | Engineer | Master | 10-19 | ＜30 |
| 28 | Senior academic | Master | 10-19 | ＜30 |
| 29 | Engineer | Master | 10-19 | 30-39 |
| 30 | Senior academic | Master | 10-19 | 30-39 |
| 31 | Engineer | Master | 20-29 | 40-49 |
| 32 | Assistant engineer | Master | 20-29 | 40-49 |
| 33 | Assistant engineer | Bachelor | 10-19 | 40-49 |
| 34 | Engineer | Bachelor | 10-19 | 30-39 |
| 35 | Assistant engineer | Master | 10-19 | 30-39 |
| 36 | Assistant engineer | PhD | 10-19 | 30-39 |
| 37 | Engineer | Master | 10-19 | 30-39 |
| 38 | Assistant engineer | Master | 20-29 | 40-49 |
| 39 | Senior academic | Master | 10-19 | 40-49 |
| 40 | Assistant engineer | Bachelor | 10-19 | 30-39 |
| 41 | Assistant engineer | Bachelor | 10-19 | 40-49 |
| 42 | Engineer | Master | 10-19 | 40-49 |
| 43 | Assistant engineer | Master | 10-19 | 30-39 |
| 44 | Engineer | Bachelor | 10-19 | 40-49 |
| 45 | Engineer | Bachelor | 10-19 | 40-49 |
| 46 | Assistant engineer | Bachelor | 10-19 | 30-39 |
| 47 | Engineer | Bachelor | 20-29 | ≥50 |
| 48 | Engineer | Master | 10-19 | 40-49 |
| 49 | Assistant engineer | Master | 10-19 | 30-39 |
| 50 | Engineer | Bachelor | 20-29 | ≥50 |
| 51 | Senior academic | Master | 6-9 | ＜30 |
| 52 | Assistant engineer | PhD | 10-19 | 30-39 |
| 53 | Assistant engineer | PhD | 20-29 | 40-49 |
| 54 | Senior academic | Master | 20-29 | 40-49 |
| 55 | Senior academic | Master | 20-29 | 40-49 |
| 56 | Senior academic | Master | 6-9 | ＜30 |
| 57 | Engineer | Master | 10-19 | 30-39 |
| 58 | Engineer | Bachelor | 6-9 | ＜30 |
| 59 | Engineer | Bachelor | 20-29 | 40-49 |
| 60 | Senior academic | Master | 20-29 | 40-49 |
